# Energy Drinks Consumption Associated with Emotional and Behavioural Problems via Lack of Sleep and Skipped Breakfast among Adolescents

**DOI:** 10.3390/ijerph18116055

**Published:** 2021-06-04

**Authors:** Zuzana Dankulincova Veselska, Daniela Husarova, Michaela Kosticova

**Affiliations:** 1 Department of Health Psychology and Research Methodology, Faculty of Medicine, P.J. Safarik University in Kosice, Trieda SNP 1, 04011 Kosice, Slovakia; daniela.husarova@upjs.sk; 2 Institute of Social Medicine and Medical Ethics, Faculty of Medicine, Comenius University in Bratislava, Sasinkova 2, 81372 Bratislava, Slovakia; michaela.kosticova@fmed.uniba.sk

**Keywords:** energy drinks consumption, sleep, breakfast consumption, emotional and behavioural problems, adolescence

## Abstract

The aim of our study was to explore whether energy drink consumption is associated with both emotional and behavioural problems and whether this association might be mediated by amount of sleep and breakfast consumption among adolescents. The nationally representative Health Behaviour in School-aged Children (HBSC) study, realised in 2018 in Slovakia in schools, was used to acquire needed data, with the research sample of 8405 adolescents from 11 to 15 years old (mean age = 13.43; 50.9% boys) who completed the questionnaires on their own in a presence of researchers and research assistants. Emotional and behavioural problems were assessed by a Strengths and Difficulties Questionnaire, while energy drinks consumption, breakfast consumption and sleep duration was assessed by questions in line with the HBSC study protocol. Linear regression models assessed the associations between energy drinks consumption and emotional and behavioural problems. Mediation by sleep duration and breakfast consumption was assessed with parallel mediation models. Energy drink consumption was significantly associated with emotional (*p* < 0.001) and behavioural problems (*p* < 0.001), with higher consumption of energy drinks leading to more emotional and behavioural problems. Results from a parallel mediation analysis indicated that energy drink consumption is indirectly related to both emotional and behavioural problems through its relationship with the amount of sleep and breakfast consumption. Parents and professionals working with adolescents should be aware that unhealthy dietary habits and lack of sleep might be related to emotional and behavioural problems.

## 1. Introduction

Emotional and behavioural problems, which occur mainly in childhood and adolescence, affect approximately 10–25% of child and adolescent populations [1,2] can have long-lasting consequences not only for adolescents but also for their families and society as a whole [3]. It is therefore of great importance to reveal factors associated with the increased likelihood of emotional and behavioural problems.

Previous research has confirmed that regular energy drink (hereinafter referred to as EDs) consumption in adolescents might be considered as one of those potential risk factors, as it has been associated with a wide range of emotional and behavioural problems including depression, stress, anxiety, emotional difficulties, self-destructive, violent and risky behavior [4,5,6,7,8,9]. These adverse effects are related mainly to the consumption of a high amount of caffeine, which can exceed 500 mg in some EDs, whereas the safe dose is 200 to 300 mg [10,11]. Caffeine, a main ingredient in EDs, is the most commonly used psychoactive drug in the world, legally and is easily available for children [4], with negative influence on their physical and mental health status [12]. Moreover, the toxic effect of caffeine is potentiated by other ingredients in EDs, such as taurine [13], guarana and additives containing caffeine, including kola nut, yerba mate and cocoa. However, the amount of caffeine in these ingredients is not required to be listed on the ED packaging labels, thus, the actual caffeine amount is often higher than listed [14,15,16]. Consumption of energy drinks is popular among adolescents and has increased in the last 10 years [11,17,18]. Evidence shows that 12 to 35% of adolescents consume energy drinks at least once every week and that consumption is more frequent among males and older adolescents [6,9,19,20].

High ED intake is also associated with unhealthy behavior, such as reduced and insufficient sleep and breakfast skipping in adolescents [7,17,21,22,23,24]. Evidence shows that 30 to 50% of adolescents sleep less than recommended 8 h [25,26,27], and the prevalence of sleep problems varies between 10 to 20% in adolescents [28,29,30]. According to the large studies conducted in Europe and other countries [31,32,33,34,35], a high proportion of adolescents were classified as breakfast-skippers, ranging from 40–50% (China, Austria, Slovenia) to 10–20% (Spain, Poland, Japan). Moreover, it has been confirmed that adolescents with insufficient sleep duration and poor sleep quality suffered from more emotional and behavioural problems [31,36,37,38,39]. Some studies have also found an association between breakfast omission and emotional and behavioural health problems, however, the quality and strength of the evidence was weak [7,31,40,41] and research indicates that the quality of breakfast might play an important role in this association [42]. Nevertheless, it remains unclear what role might amount of sleep and breakfast consumption play in the association between ED consumption and emotional and behavioural problems. Our study provides the opportunity to add to the existing knowledge by exploring one of the potential pathways between EDs and emotional and behavioural problems via the amount of sleep and breakfast consumption on a nationally representative sample.

Therefore, the aim of our study was to explore whether ED consumption is associated with both emotional and behavioural problems and whether this association might be mediated by the amount of sleep and breakfast consumption among adolescents.

## 2. Materials and Methods

### 2.1. Sample and Procedure

To explore our aim, we used data from the nationally representative Health Behaviour in School-aged Children (HBSC) study realised in 2018 in Slovakia that is part of World Health Organisation collaborative, cross-national HBSC study of 11-, 13- and 15-year-old school-aged children from 50 countries and regions across Europe and North America. The study focused on health and health-related behaviours in their social context, with the aim to deepen the understanding of the mechanisms influencing differences and changes in the health and health-related behaviour of school-aged children [42]. More detailed information about the two-step sampling process and procedure used to acquire this nationally representative sample could be found in our previously published paper [26]. On the school level, we approached 140 randomly chosen schools from all regions in Slovakia (in rural as well as urban areas) with a response rate 77.9%. On an individual level, we collected questionnaires in Slovak (and in case of schools with a Hungarian minority, in Hungarian) language from 8405 adolescents from 11 to 15 years old (mean age 13.43; 50.9% boys). Pupils completed the self-reported questionnaires at school on their own, in the presence of researchers and research assistants.

### 2.2. Ethics

The study was approved by the Ethics Committee of the Medical Faculty at the P.J. Safarik University in Kosice (16N/2017). First, schools were contacted and were asked for participation in the study on a voluntary basis. Second, parents were informed about the study via the school administration and could opt-out if they disagreed with their child’s participation. Finally, pupils themselves were given the opportunity to not participate in a data collection even if their parents provided the consent. Participation in the study was therefore fully voluntary and anonymous, with no explicit incentives provided for participation on all levels of data collection.

### 2.3. Measures

To cover energy drinks consumption, we asked the question: “How many times a week do you usually drink energy drinks, for example, Red Bull?” with answers: (1) “never”; (2) “less than once a week”; (3) “once a week”; (4) “2–4 days a week”; (5) “5–6 days a week”; (6) “once a day, every day” and (7) “every day, more than once” [8].

To cover breakfast consumption, we asked the question: “How often do you usually have breakfast during weekdays (more than a glass of milk or fruit juice)?” with answers: (1) “I never have breakfast during a week”, (2) “one day”, (3) “two days”, (4) “three days”, (5) “four days” and (6) “five days” [42].

To cover sleep duration, we computed the time between bedtime and wake-up time on school days. We covered bedtime and wake-up time with two questions: “When do you usually go to bed if you have to go to school the next morning?” and “When do you usually wake up on school mornings?” with answers ranging in half-hour intervals [42,43].

Emotional and behavioural problems were measured with the Strengths and Difficulties Questionnaire (SDQ). This questionnaire consists of 25 items [44], of which we used the 20 items covering emotional and behavioural problems. Answers were: not true (0), somewhat true (1), and certainly true (2). We calculated emotional (internalising) problems subscale (score 0–20) and behavioural (externalising) problems subscale sum score (score 0–20) [45]. A higher score indicates more problems. We explored Cronbach’s alpha which was 0.73 for the whole scale and 0.71 for the emotional and behavioural problems subscales, respectively.

Family affluence was measured using the Family Affluence Scale III (FAS-III), which consists of six questions: “Does your family own a car, van or truck?” (No/Yes, one/Yes, two or more), “Do you have your own bedroom for yourself?” (Yes/No), “How many computers does your family own?” (None/One/Two/More than two), “How many bathrooms (room with a bath/shower or both) are in your home?” (None/One/Two/More than two), “Does your family have a dishwasher at home?” (Yes/No), “How many times did you and your family travel out of your country for a holiday/vacation last year?” (Not at all/Once/Twice/More than twice). We computed the sum score, which we converted to a ridit score ranging from 0 to 1. We then created tertile categories of low (0 to 0.333), medium (0.334 to 0.666) and high (0.667 to 1) socioeconomic position [46].

### 2.4. Statistical Analysis

**Figure 1:**
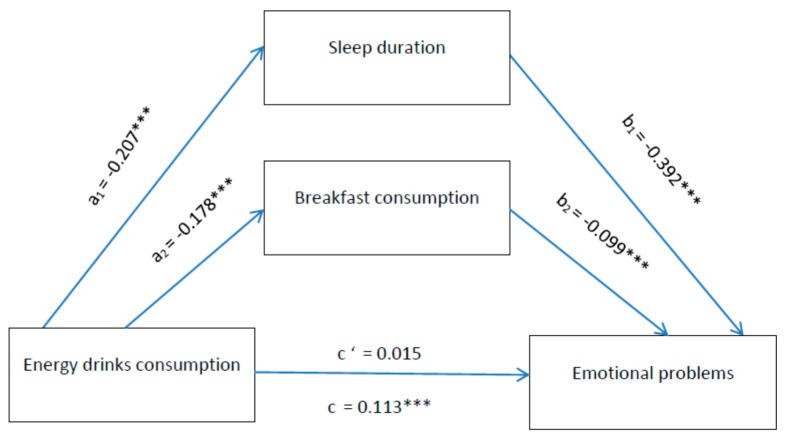
Mediation by sleep duration and breakfast consumption of the relation between EDs and emotional problems. Notes: *** *p* < 0.001. All presented effects are unstandardised; a_n_ is the effect of ED consumption on mediators; b_n_ is the effect of mediators on emotional problems; c’ is the direct effect of ED consumption on emotional problems, and c is the total effect of ED consumption on emotional problems.

**Figure 2:**
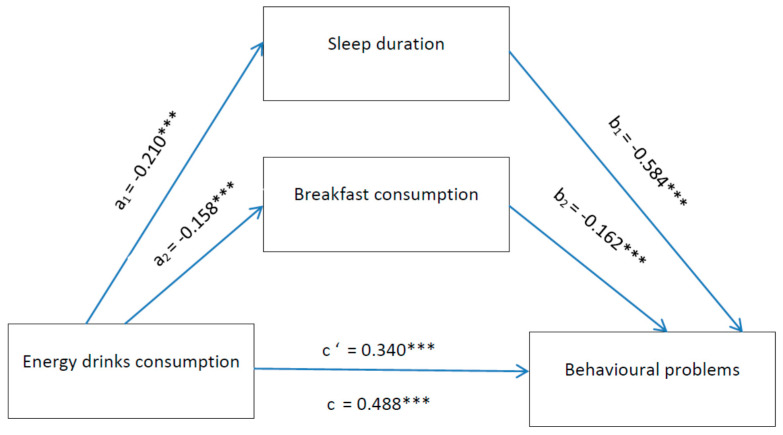
Mediation by sleep duration and breakfast consumption of the relation between EDs and behavioural problems. Notes: *** *p* < 0.001. All presented effects are unstandardised; a_n_ is the effect of ED consumption on mediators; b_n_ is effect of mediators on behavioural problems; c’ is the direct effect of ED consumption on behavioural problems, and c is the total effect of ED consumption on behavioural problems.

First, we described the background characteristics of the sample using descriptive statistics. Second, we performed a series of analyses to explore the associations of ED consumption, sleep duration and breakfast consumption with emotional and behavioural problems, using linear regression analysis. We repeated these analyses with adjustment for gender, age, and family affluence. Next, we conducted a final analysis on the parallel mediation by sleep duration and breakfast consumption of the relation between ED consumption and emotional and behavioural problems. We did so by assessing the mediation effect of all variables separately and then building a parallel mediation model using the PROCESS macro model 6 [30]. These analyses were all controlled for gender, age and family affluence, and all indirect effects were subjected to follow-up bootstraping analyses, with 5000 bootstrap samples. The indirect effect was calculated using the a*b product method, and bootstrapped 95% confidence intervals for the indirect effect of ab was provided as a test of the indirect effect [47]. Arrows were added to the parallel mediation models in Figure 1 and Figure 2 to illustrate the mediation, expected and tested direction of the associations presented. All analyses were performed in SPSS v. 23 for Windows (IBM Corporation, New York, NY, USA).

## 3. Results

### 3.1. Baseline Characteristics

Table 1 and Table 2 shows the basic descriptive statistics of the studied variables in our research sample.

### 3.2. Associations of Energy Drinks Consumption with Emotional and Behavioural Problems Mediated by Sleep Duration and Breakfast Consumption

In the exploratory linear regression analyses presented in Table 3, we found significant association between ED consumption and both emotional and behavioural problems; a higher frequency of ED consumption was positively associated with both emotional and behavioural problems. We also found both assumed mediators to be significantly associated with both emotional and behavioural problems; higher amount of sleep and higher frequency of breakfast consumption were negatively associated with both emotional and behavioural problems even after adjustment for gender, age and perceived socioeconomic status of the family (Model 1). Association with emotional problems lost its significance after adding sleep duration and breakfast consumption, association with behavioural problems decreased but remain significant after adding sleep duration and breakfast consumption (Model 2).

Results from a parallel mediation analysis indicated that ED consumption was indirectly related to both emotional and behavioural problems through its relationship with amount of sleep and breakfast consumption. As can be seen in Figure 1, we found that adolescents with higher ED consumption reported lower amount of sleep and lower frequency of breakfast consumption. The lower amount of sleep and lower frequency of breakfast consumption was subsequently related to more emotional problems, respectively. A 95% bias-corrected confidence interval based on 5000 bootstrap samples indicated that the indirect effects through sleep duration and breakfast consumption was entirely above zero), and therefore significant.

We found similar findings also for behavioural problems, as could be seen in Figure 2. Adolescents with higher EDs consumption reported lower amount of sleep and lower frequency of breakfast consumption. A lower amount of sleep and lower frequency of breakfast consumption was subsequently related to more behavioural problems, respectively. A 95% bias-corrected confidence interval based on 5000 bootstrap samples indicated that the indirect effects through sleep duration and breakfast consumption was entirely above zero, therefore significant.

## 4. Discussion

The aim of our study was to explore whether ED consumption is associated with emotional and behavioural problems and whether this association might be mediated by the amount of sleep and breakfast consumption among adolescents.

We found in our analysis that higher consumption of ED was associated with both emotional and behavioural problems. Our results are consistent with the results of other studies. Adolescents who regularly drink EDs reported more emotional difficulties and symptoms of depression, anxiety, nervousness and stress [6,7,9,48,49]. There is also evidence that the consumption of EDs among adolescents is linked to increased risk of wide range of negative behavioural outcomes such as substance use, binge drinking, aggressive, violent and self-destructive behavior, hyperactivity/inattention symptoms and sensation seeking behavior [5,8,11,18,19,49]. The adverse effects of EDs on emotions and behavior of adolescents can be explained by the consumption of high doses of caffeine [12] and other psychoactive agents such as taurine, guarana or ginseng [4,11]. Energy drinks therefore pose potential health risks when it comes to emotions and behaviours because of above mentioned stimulants content, and are inappropriate for adolescents [10]. However, adolescents are not aware about these potential risks, and on the contrary, they have expectations about the positive effects of EDs on mood, performance and alertness [17]. Moreover, they consider EDs as an easy source of energy that helps them to cope with stressful situations and experience positive emotions such as pleasure and excitement [11]. Although the evidence shows a positive association between the consumption of EDs and emotional and behavioural problems in adolescents, it is not clear whether this relationship is casual and what and how other factors might influence this association. We therefore examined the role of sleep duration and breakfast on the association between ED consumption and emotional and behavioural problems in adolescents.

We found that sleep duration mediated the association between EDs consumption and both emotional and behavioural problems among adolescents. Adolescents who consumed a higher amount of EDs slept less and reported more emotional and behavioural problems. Previous studies confirmed our findings that excessive use of EDs was associated with reduced and insufficient sleep duration [20,21,23,50] or even insomnia [24]. The association between insufficient sleep duration and emotional and behavioural problems has also been confirmed by several cross-sectional, experimental studies, as well as by systematic reviews [25,26,37,38,39,51,52,53]. Although most of the studies were cross-sectional and used subjective measures for sleep duration, the evidence from the experimental studies and systematic reviews suggests that the relationship between lack of sleep and emotional and behavioural problems in adolescents is likely to be casual and thus supports our findings that the effect of ED consumption on emotional status and behaviour of adolescents is not direct, but mediated through sleep duration. Furthermore, the association between sleep duration and emotional and behavioural problems is likely to be bidirectional and more complex, with other factors contributing to this association [51,53]. In our study, we also focused on one aspect related to the dietary habits of adolescents, specifically on the frequency of breakfast consumption, whose mediating role on the association between EDs consumption and emotional and behavioural problems will be discussed next.

Finally, we found that breakfast consumption mediated the association between ED consumption and both emotional and behavioural problems among adolescents. Adolescents who consumed a higher amount of EDs regularly skipped breakfast during weekdays and reported more emotional and behavioural problems. We have found just two studies focusing on the relation between EDs and breakfast consumption and their impact on mental health and behaviour of adolescents [7,22]. The studies have provided evidence that breakfast omission was associated with stress, anxiety and depression levels and poor academic performance in adolescents, and that this was largely observed for both those who frequently consumed EDs and those who did not. Energy drink consumption was associated with more frequent consumption of junk food and breakfast omission. However, it is important to notice that other factors might be responsible for breakfast skipping in addition to the ED consumption, e.g., absent family rules regarding breakfast consumption. The findings indicate that consumption of EDs is associated with negative mental and behavioural outcomes not directly, but rather being part of an unhealthy diet, including breakfast skipping.

### 4.1. Strengths and Limitations

The major strength of our study is its large nationally representative sample and the use of validated measures as a part of a standard HBSC questionnaire in the confidential setting, with children filling out the anonymised questionnaires in the presence of a research assistant only. Some limitations regarding our study should be mentioned as well. First, the cross-sectional design limits our ability to establish causal relationships. It cannot be determined if ED consumption contributes to the poor mental health of young people as it is possible that young people may use energy drinks to manage symptoms related to poor mental health (such as feeling tired or poor concentration). Second, we used self-reported measures, which are not able to measure sleep and EDs and breakfast consumption as precisely. This issue, combined with recall bias, may influence the accuracy of data, with the possibility of over and underreporting. Nevertheless, this is common in questionnaire studies that cover a broad range of topics and a large sample of participants, such as the HBSC study, and previous research has shown them to be valid [26,54,55]. Third, the sleep duration was calculated as a difference between wake-up time and bedtime, which is the time spent in bed and not the time spent asleep. This could have caused the number of sufficient sleepers to be overestimated.

### 4.2. Implications

Professionals working with children should be aware that unhealthy dietary habits and lack of sleep might be related to emotional and behavioural problems in adolescents. They should consider that consumption of high amounts of EDs, breakfast skipping and lack of sleep may serve as markers to identify adolescents at risk for emotional and behavioural problems [4,8,9]. They should educate parents and adolescents about the importance and health benefits of regular breakfast consumption and sufficient sleep duration based on existing international recommendations [56] and recommend avoiding the consumption of EDs. Public health strategies should focus on the restriction of EDs consumption among children and adolescents by the limitations of availability of Eds, including the implementation of age limits in stores and sales ban in schools [5,10,11]. To formulate the strategies and intervention to promote adolescents’ health behaviour and well-being, more emphasis should be put on regular breakfast consumption and sufficient sleep duration. Further research is needed to examine the other factors influencing the relationships between ED consumption and emotional and behavioural problems of adolescents. To establish the causality in relationships, longitudinal and randomised controlled studies are required to be conducted.

## 5. Conclusions

Adolescents with higher consumption of EDs reported more emotional and behavioural problems in line with previously published findings. Our study provides additional information about one of the possible ways such associations might be maintained. We revealed that the associations between EDs and emotional and behavioural problems were mediated by the amount of sleep and breakfast consumption. These findings suggest that parents and professionals working with adolescents should be aware that unhealthy dietary habits and lack of sleep might be related to emotional and behavioural problems.

## Figures and Tables

**Table 1:** Descriptive statistics of the sample (HBSC-study, Slovakia 2018, 11–15 years old, N = 8405).

Variables	Total
	N = 8405
Gender (N, %)	
Boys	4282 (50.9%)
Age (mean, SD)	13.43 (±1.33)
FAS-III (mean, SD)	7.76 (±2.48)
Emotional and behavioural problems sum score (mean, SD)	
Emotional problems	15.13 (±3.33)
Behavioural problems	16.49 (±3.26)

HBSC-study, Health Behaviour in School-Aged Children study, N, number of respondents, FAS-III, Family Affluence Scale III, SD, standard deviation.

**Table 2:** Frequencies of everyday energy drinks consumption, everyday breakfast consumption and sufficient sleep duration stratified by age and gender (HBSC-study, Slovakia 2018, 11–15 years old, N = 8405).

	Girls			Boys		
	11-Years (N, %)	13-Years (N, %)	15-Years (N, %)	11-Years (N, %)	13-Years (N, %)	15-Years (N, %)
Everyday energy drinks consumption	49 6.1%	96 10.9%	103 18.1%	99 13.2%	200 21.3%	207 31.2%
Everyday breakfast consumption	382 48.2%	363 40.7%	210 36.5%	405 54.1%	478 50.4%	289 43.2%
Sleep duration 8 or more hours	686 85.6%	597 65.5%	289 49.7%	642 86.1%	675 70.2%	363 53.1%

**Table 3:** Associations between ED, sleep duration and breakfast consumption with emotional and behavioural problems based on linear regression analysis leading to regression coefficients (B) adjusted for gender, age and family affluence (HBSC-study, Slovakia 2018, 11–15 years old, N = 8405).

	Emotional Problems		Behavioural Problems	
	Model 1 B	Model 2 B	Model 1 B	Model 2 B
Energy drinks consumption	0.11 ***	0.02	0.49 ***	0.34 ***
Sleep duration	−0.43 ***	−0.39 ***	−0.73 ***	−0.58 ***
Breakfast consumption	−0.13 ***	−0.10 ***	−0.23 ***	−0.16 ***

Notes: *** *p* < 0.001; Model 1, univariate analysis adjusted for gender, age and family affluence; Model 2, multivariate analysis adjusted for gender, age and family affluence.

## Data Availability

The data presented in this study are available on request from the corresponding author. The data are not publicly available due to privacy and ethical issues.
